# Development and Application of the Agricultural Product Safety Index in Major Countries and Imported Food Safety Index for Korea

**DOI:** 10.3390/foods14142461

**Published:** 2025-07-14

**Authors:** Da-Eun Jung, Sung-Bum Yang

**Affiliations:** Department of Food and Resource Economics, Dankook University, Cheonan 31116, Republic of Korea; smiler@dankook.ac.kr

**Keywords:** agricultural products safety index, imported food safety index, sustainable food systems, sustainable food production, food safety

## Abstract

With the growth of international trade, concerns over the safety of imported agricultural products in South Korea have intensified due to factors such as the COVID-19 pandemic, radiation contamination risks, and the prevalence of GMOs. In response, this study develops two composite indices—the Agricultural Product Safety Index (APSI) and the Imported Food Safety Index (IFSI)—to quantitatively assess food safety risks across major exporting countries and apply them to Korea’s import structure. The indices integrate production and distribution risk indicators based on publicly available data and adhere to five key principles, including applicability, reliability, boundedness, independence, and representativeness. Empirical results from 2014 to 2021 indicate that Australia consistently demonstrates the highest food safety level, followed by the United States, Argentina, Ukraine, and Brazil. While the indices provide a structured and transparent framework for monitoring import-related safety, their scope is limited to selected countries and excludes biological hazards due to data limitations. Future research should expand the geographical coverage and incorporate empirical validation techniques. These findings contribute to the development of evidence-based policy instruments aimed at enhancing food safety governance in global supply chains.

## 1. Introduction

With the expansion of international trade, the volume of imported agricultural and food products in South Korea has been steadily increasing. However, growing concerns over food safety have emerged due to factors such as the COVID-19 pandemic, risks of radiation contamination, and the importation of genetically modified organisms (GMOs). These concerns have intensified public demand for a more systematic and proactive approach to food safety.

In response, South Korea enacted the Special Act on Imported Food Safety Control in February 2016 and established the Food Safety Policy Bureau under the Ministry of Food and Drug Safety in March 2017. These developments laid a legal and administrative foundation for food safety. However, the current system still relies heavily on post-import inspections and faces limitations in addressing emerging risks such as infectious diseases, climate change, and environmental pollutants. Merely meeting legal standards is no longer sufficient to build consumer trust. There is a growing need for a forward-looking, trust-based safety management system grounded in scientific evaluation. In particular, the development of quantitative safety indicators would enable more effective risk assessments, informed policy decisions, and improved public communication.

Globally, several countries and international organizations have implemented food safety initiatives. Codex Alimentarius provides international standards, while the U.S. FDA’s Food Safety Modernization Act (FSMA) promotes preventive controls, including the Foreign Supplier Verification Program (FSVP). The European Union operates the Rapid Alert System for Food and Feed (RASFF) and enforces preventive standards based on Hazard Analysis and Critical Control Points (HACCP). Japan has established residue standards for pesticides and additives in imported food products.

Additionally, two notable indices provide food safety-related assessments: the Global Food Security Index (GFSI), published by the Economist Group, and the Food Import Vulnerability Index (FIVI), developed by the International Food Policy Research Institute (IFPRI). The GFSI covers 113 countries and includes a “Quality and Safety” sub-index that evaluates the enabling environment for food safety, comprising factors such as relevant legislation, monitoring mechanisms, access to drinking water, and storage conditions. However, this index does not specifically focus on the safety of imported foods. The FIVI assesses vulnerabilities related to imports of 15 major food commodities across 182 countries, with indicators such as caloric import share, import dependence, and food insecurity prevalence. While informative, the FIVI does not explicitly account for food safety factors and remains in an early stage of methodological refinement.

While existing indices such as the Global Food Security Index (GFSI) and the Food Import Vulnerability Index (FIVI) offer valuable perspectives, they fall short of directly addressing the safety of imported food products [1]. For example, the FIVI, developed by the International Food Policy Research Institute (IFPRI), is designed to assess a country’s vulnerability to food supply shocks based on caloric import dependency, trade diversification, and the prevalence of food insecurity. Although useful for identifying structural weaknesses in food supply chains, the FIVI does not include indicators related to food safety risks, such as biological contamination, pesticide exposure, or distribution-level integrity. It is primarily intended to guide nutritional security and supply risk policy, rather than serve as a tool for evaluating contamination-related food safety hazards. In contrast, the indices proposed in this study—the Agricultural Product Safety Index (APSI) and the Imported Food Safety Index (IFSI)—are explicitly designed to quantify the relative safety of imported agricultural products by integrating both production- and distribution-stage risk indicators.

Previous studies have highlighted the importance of legal and institutional infrastructure in enhancing food safety [2,3,4,5]. For example, Jongwanich [2] analyzed the effects of food safety regulations on processed food imports in developing countries, emphasizing the role of agricultural supply capacity. Choi et al. [6] proposed an analytical method to evaluate imported agricultural products’ safety, while Welburn et al. [7] assessed import risks using normalized import refusals. Lee et al. [8] investigated the link between consumer food safety perception and trust in policy, and Yang [9] constructed time-series safety indices using value chain data. Although informative, these studies lack a comprehensive, integrated index to evaluate import-related food safety risks across multiple countries and contexts.

To fill this gap, this study develops two complementary indices: the Agricultural Product Safety Index (APSI), which assesses the food safety performance of major exporting countries, and the Imported Food Safety Index (IFSI), which applies the APSI framework to Korea’s food import structure. These indices are constructed using both production and distribution risk indicators and are intended to support proactive food safety management, evidence-based policymaking, and improved consumer confidence.

Throughout the manuscript, we consistently use the terms APSI and IFSI to distinguish between the global safety performance assessment and its application to Korea’s import profile. This study ultimately aims to offer a robust, replicable framework for evaluating and enhancing food safety in the context of globalized trade.

## 2. Methodological Framework

### 2.1. Analytical Model

This study aims to quantify the safety of imported agricultural products by country and year, thereby providing foundational data to inform future policy development. Accordingly, the proposed index targets major importing countries selected based on the Concentration Ratio (CR3), and consists of two annual sub-indicators.

To systematically assess the safety of agricultural products across countries and over time, this study establishes five key principles as outlined in Table 1 [10]. 1. Applicability: The index should allow for quantitative comparisons across countries and time periods. 2. Reliability: The index must exclude estimates derived from proprietary sources or modeling techniques inaccessible to external reviewers. It must also ensure reproducibility by using publicly available and credible data. To reinforce methodological transparency, all equations were constructed using national-level data from open international databases. This approach facilitates consistent replication of results and supports broader policy applications. 3. Boundedness: The index is defined on a scale from 0 to 1, where values closer to 1 indicate higher levels of safety [11]. 4. Independence among indicators: Each indicator should be independent and free of redundancy. 5. Representativeness: The index is calculated using countries selected each year based on the CR3 criterion.

**Figure 1:**
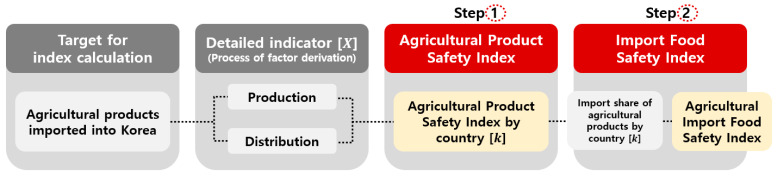
Flowchart of the Agricultural Product Safety Index calculation.

The analytical model comprises a two-step process (Figure 1). Step 1 calculates the Agricultural Product Safety Index (APSI) for each exporting country annually, using a set of detailed production and distribution indicators.

Each composite indicator is derived by averaging relevant sub-indicators. Step 2 computes the Imported Food Safety Index (IFSI) by weighting the APSI scores of individual countries according to Korea’s annual import proportions.

**Figure 2:**
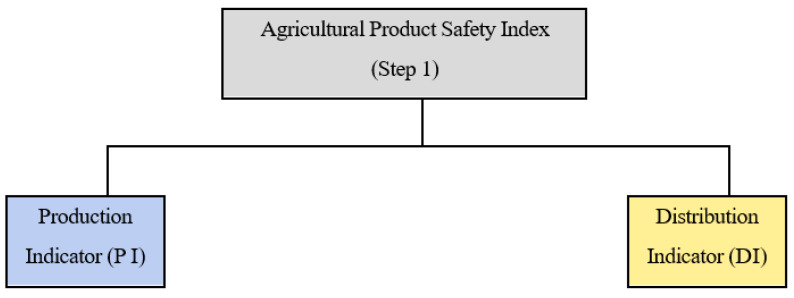
Classification of sub-indicators in the Agricultural Product Safety Index.

To ensure that the indices reflect the full spectrum of food safety risks, the model considers risk factors spanning from production in the country of origin to the point of import. Accordingly, indicators are categorized into Production Indicators (PI) and Distribution Indicators (DI) (Figure 2). Risk factors include both biological (e.g., pathogens) and chemical hazards (e.g., environmental pollutants and food additives) [8,9]. Climate variability, stock levels, government investment in agriculture, trade distance, and political stability were also identified as key determinants affecting food safety in international trade contexts.

Accordingly, the following sub-detailed indicators were selected under production and distribution indicators based on chemical, environmental, and other factors (Table 2).

The ‘production indicator’ represents the level of safety in the agricultural production stage of the exporting country and consists of seven sub-indicators. In this study, abnormal rainfall events (e.g., drought and flood) were considered as environmental factors, and farm product diseases (e.g., pests) as biological factors. However, these two sub-indicators were excluded from the indexing process due to data limitations. For chemical factors, the following sub-indicators were selected considering pesticide and heavy metal use: ‘pesticide use per hectare of agricultural land’, ‘fertilizer use per hectare of agricultural land’, and ‘quality of agricultural water’.

For environmental factors, ‘temperature anomalies (including both high and low temperatures)’ and ‘acid rain’ were selected as sub-indicators. Other factors included the ‘stock levels’ of agricultural products in the country and ‘governmental investment in the agricultural sector’. The ‘distribution indicator’ refers to the level of food safety during the process of importing agricultural products into Korea from abroad. As sub-indicators, this study considered the trade ‘distance from the importing country to Korea’ as a factor that may influence food safety. It also judged the ‘import non-compliance rate’ to be a direct indicator for evaluating the level of food safety. Finally, the study defined the ‘political stability’ of the exporting country as an indirect indicator that could affect food safety.

To operationalize this index framework, the authors formulated new mathematical expressions to calculate the Agricultural Product Safety Index (APSI) and the Imported Food Safety Index (IFSI). These equations were originally developed for this study and are not derived from existing models in the prior literature. This design choice ensures that the index structure aligns with the five principles outlined above—particularly transparency, reproducibility, and boundedness—while maintaining consistency with the selected sub-indicators and publicly available data sources.

Each of the sub-detailed indicators is calculated in the three following ways depending on the type of observed values: Calculation Method 1: If the detailed indicator X as a continuous variable meets the boundedness condition of 0≤X≤1, that indicator is used as it is. Calculation Method 2: When a continuous variable satisfies the boundedness condition but its minimum value is below 0 or its maximum exceeds 1, the full range is divided into 100 equal intervals, and evaluation scores from 0.01 to 1.00 are assigned accordingly. Calculation Method 3: If the continuous variable fails to meet the boundedness condition, the value is calculated using Equations (1) and (2) below (Equations (1) and (2) are methods applicable to calculating detailed evaluation indicators of the index presented in [12,13].):

(1) $$\text{Indicator}\;X_{k,t}=\frac{X_{k,t}-X_{\text{min}}}{X_{\text{max}}-X_{\text{min}}}\;\;“\text{The larger the value},\text{the better}”\;$$

(2) $$\text{Indicator}\;X_{k,t}=1-\frac{X_{k,t}-X_{\text{min}}}{X_{\text{max}}-X_{\text{min}}}\;\;“\text{The smaller the value},\text{the better}”$$

X_max_: maximum value of X in all of the measurable countries (k) and during the entire period (t)
X_min_: minimum value of X in all of the measurable countries (k) and during the entire period (t)

To ensure transparency and replicability, all sub-indicators for both production and distribution dimensions are aggregated using a simple arithmetic mean. This approach avoids the use of arbitrary or subjectively assigned weights, thereby allowing other researchers to reproduce the results using the same dataset. This method is consistent with best practices in composite index development, where equal weighting is often used to avoid the subjectivity or opaqueness introduced by statistically derived weights, particularly in exploratory models designed for transparency and comparison [14].

In Step 1, the Agricultural Product Safety Index (APSI) for each exporting country is calculated as the arithmetic average of its sub-indicators, as illustrated in Figure 1 and expressed in Equation (3). In Step 2, the Imported Food Safety Index (IFSI) for Korea is computed by weighting each country’s APSI score by its respective share of Korea’s total agricultural imports. This calculation is based on the three countries (CR3 refers to the Cumulative Ratio of the top 3 exporting countries based on their share in Korea’s total import volume [15].) with the highest cumulative share in Korea’s import structure, as shown in Equation (4). A higher index value indicates a higher level of food safety for agricultural products imported from the corresponding country.

(3) $$\text{Step}\;1:\text{Agricultural Product Safety Index}\;=\;\frac{1}{2}\left(PI\;+\;DI\right)$$

(4) $$\text{Step}\;2:\text{Imported Food Safety Index}\;=\;\sum_{k=1}^{K}({\text{Import}\;\text{share}}_{k}\times {\text{Agricultural Product Safety Index}}_{k})$$

Import share_k_: calculated in two ways according to the K
K: representative countries selected based on CR3 criteria

The formula adopts a basic weighted arithmetic structure, consistent with established index construction methodologies such as those used in the Global Food Security Index (GFSI), the Food Import Vulnerability Index (FIVI), and the Human Development Index (HDI). This approach ensures comparability, interpretability, and transparency, while remaining tailored to the specific context of food import safety assessment.

### 2.2. Analysis Data and Indicator Calculation

The analytical data were collected from authoritative sources that are accessible by anyone according to the second principle (reliability that ensures replicability). Among the sub-detailed indicators of the production index, ‘pesticide use per area of cropland’ is based on [16] data (Table 3).

For continuous data that do not meet the boundedness condition and where a smaller value is considered better, the calculation is based on Equations (1) and (2) of Calculation Method 3.

In the case of ‘fertilizer use per area of cropland,’ we derived the value using [14] data on agricultural land and agricultural use fertilizers. This indicator was derived using Equations (1) and (2), as smaller values are considered better. ‘The quality of agricultural water’ refers to the ‘agricultural water risk: quality’ in the Global Food Security Index (GFSI), which is released each year by [17]. As the value is a continuous variable and meets the condition of boundedness, the index was calculated with the GFSI-released value divided by 100 as in Calculation Method 1.

For ‘the extreme temperature anomaly,’ anomaly data released by the National Oceanic and Atmospheric Administration [18] were used. NOAA reports temperature anomalies as deviations from the 1991–2020 average. In this study, after inputting the latitude and longitude of each country’s capital, the total sum of the absolute values of monthly temperature anomalies was defined and calculated as the ‘temperature anomaly’ of the year. In this case, since both increases (extreme heat) and decreases (extreme cold) in temperature anomalies are considered undesirable, the value was calculated using Equations (1) and (2). For ‘inventory levels,’ data on the ending stocks of all agricultural products by country from the U.S. Department of Agriculture [19] were used. Since lower inventory levels are considered better, the calculation was based on Equations (1) and (2). For ‘governmental investment,’ the proportion of governmental investment in agriculture and forestry was calculated in comparison with the total expenditure of the central government. To this end, data from the [17] on total central government expenditure and investment in agriculture, forestry, and fishing were used for the calculation. Since higher values are considered better, the index was calculated based on Equation (1). Finally, for ‘acid rain,’ data from the 2023 Environment Performance Index (EPI), published annually by Yale University, were used. Among the Yale EPI data, the sulfur dioxide (SDA) increase rate and the nitrogen oxides (NDA) increase rate (on the 100-point scale) were weighted at 50% to calculate the index [20]. Since the value is a continuous variable and satisfies the condition of boundedness, it was calculated by dividing the score by 100, in accordance with Calculation Method 1.

Among the sub-detailed indicators of ‘the distribution indicator,’ the ‘Bilateral Trade Distance’ was based on bilateral distance data released by the Centre d’Etudes Prospectives et d’Informations Internationales [21]. The destination country (iso_d) was set to South Korea. Since a shorter distance was deemed better, the index was calculated using Equations (1) and (2). The ‘Import Non-compliance Rate’ was calculated using data provided by [22]. We calculated values for agricultural products by country of origin (manufacturing country). Since lower values were considered to indicate better outcomes, we applied Equations (1) and (2) to compute the index. Finally, for ‘political stability,’ World Bank data were utilize [23]. The variable is continuous and bounded between a minimum value of –3.31 and a maximum value of 1.69. However, since the range falls outside the 0–1 interval, scoring was performed using Calculation Method 2. The descriptive statistics calculated accordingly are presented in Table 4.

This index was calculated based on CR3 for major countries from which agricultural products were imported (Table 5).

Accordingly, major importing countries from 2014 to 2021 were selected annually based on the volume of imports, using the data from [24]. Agricultural products were defined as those within HS Code digits 06 to 13.

Although the major importing countries varied by year, five countries—the United States, Brazil, Australia, Ukraine, and Argentina—were selected as common representatives.

## 3. Results

### 3.1. Calculation of the Agricultural Product Safety Index by Country (Step 1 Index)

**Figure 3:**
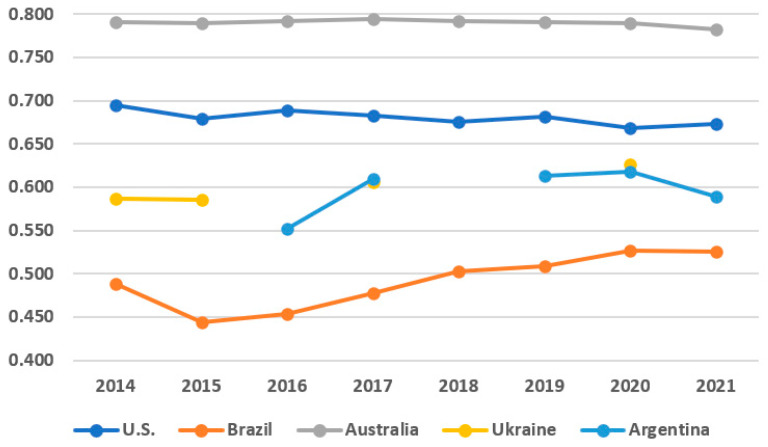
Calculation of the Agricultural Product Safety Index by country (2014–2021).

This study calculates the Agricultural Product Safety Index for 5 major importing countries (Figure 3 (Missing values of Argentina (2014–2015, and 2018) and Ukraine (2016, 2018–2019, and 2021) indicate years when they were not included among representative countries based on CR3 criteria)). Argentina was not included among the representative countries based on the CR3 criteria in 2014–2015 and 2018, while Ukraine was excluded in 2016 and 2018–2019. This explains the absence of corresponding values for these countries in the relevant figures.

Step 1 calculation results of the Agricultural Product Safety Index by country show that, from 2014 to 2021, Australia had the highest level (0.8105) on average, followed by the U.S. (0.7073), Argentina (0.6359), Ukraine (0.6272), and Brazil (0.5833) in that order. Australia’s share of agricultural product imports is relatively low among major importing countries, but over the past 8 years, its safety has been at the highest level (see Table 6).

The significant rise since 2015 (∆0.0054) turned into a declining trend starting in 2016 (∇0.0048 in 2021 compared to 2016). During the past 3 years (2019–2021), the import share was the lowest among major importing countries, but it showed a slight increase in 2021 compared to 2020 (∆1.28 percentage points). In the case of the U.S., the share of agricultural product imports was the highest over the past 8 years, and it showed the second-highest level of safety among the five representative countries (see Table 7).

Since 2014, however, the imported food safety index has shown a slight downward trend. Notably, in 2020, the decline was significant (∇0.0140) compared to the index in 2018. While its import share is the largest among the five countries, it showed a 19.54 percentage point decrease in 2019 compared to 2018, and over the past three years (2019–2021), it has been at the lowest level in the entire period. Over the past 3 years (2019–2021), Argentina maintained an average level of 0.6473 and showed the third-highest food safety level among representative countries. Notably, in 2017, it showed a significant increase compared to 2016 (0.5894 → 0.6482, ∆0.0588), maintaining a similar level over the past 3 years (Table 8).

Regarding import share, between 2020 and 2021, Argentina had the second-largest import share among representative countries.

Specifically, in 2021, it showed a significant increase from the previous year (∆7.12 percentage points), accounting for the second-largest share, just behind the U.S. Ukraine maintains a similar food safety level to that of Argentina (Table 9), and it has shown an upward trend since 2014. Notably, in 2020, it showed a significant increase compared to 2014 (0.6147 → 0.6454, ∆0.0307). However, its import share has remained at a relatively low level, averaging 9.85% over the past 4 years.

Brazil has shown the lowest food safety level among representative countries, below 0.6000 over the past 8 years. In 2020, however, its level increased significantly compared to 2019 (0.5733 → 0.5963, ∆0.0230).

Most recently, its level has been the highest over the entire period (0.5959) (Table 10). Its import share increased significantly in 2019 compared to 2018 (∆12.36 percentage points), but over the past 3 years, it has shown a continuous downward trend (∆7.52 percentage points in 2021 compared to 2019).

### 3.2. Calculation of the Agricultural Product Safety Index for Korea (Step 2 Index)

**Figure 4:**
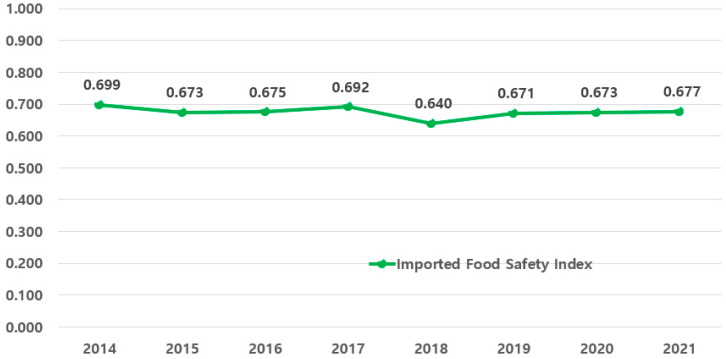
Calculation of the Agricultural Product Safety Index (2014–2021).

In this section, the safety level of imported agricultural products by year is calculated based on the imported food safety index to Korea (Step 1 index) of representative agricultural import countries and their import shares (Figure 4). The analysis results show that a similar safety level has been maintained over the past 8 years.

As the index was calculated based on the import share, the average over the past 8 years was 0.675. Similar to the previous analysis, there was a significant decline in 2018 compared to 2017 (∇0.0523), but it has shown a gradual upward trend to 2021 (Table 11).

## 4. Discussion

This study focused on developing the Agricultural Product Safety Index (APSI), a quantitative tool for evaluating the safety of imported food products, and applying it to Korea’s agricultural imports by country. The index was designed to provide a multidimensional assessment of international food import risks and to support regulatory planning toward a more sustainable agricultural system and a resilient safety framework.

The findings of this study are as follows. First, the APSI establishes a structured and quantitative system for comparing the safety of imported agricultural products across countries and years, based on five key principles: applicability, reliability, boundedness, indicator independence, and representativeness. To account for risks at both the production and import stages, seven sub-indicators were selected and calculated using authoritative, open-source data.

Second, the safety index was applied to major exporting countries identified using the CR3 method, based on Korea’s import volumes from 2014 to 2021. Australia demonstrated the highest safety level, followed by the United States, Argentina, Ukraine, and Brazil. These differences reflect the influence of various factors such as agricultural practices, trade procedures, and political stability. For instance, Australia’s consistently high APSI scores can be attributed to its strong regulatory frameworks for pesticide use, efficient water management systems, and institutional stability. In contrast, Brazil’s relatively low values may reflect higher pesticide intensity, limited environmental oversight, and weaker transparency in food governance. These findings suggest that the APSI effectively captures systemic risk differentials across countries and can support risk-based import prioritization and targeted regulatory responses.

Third, Korea’s overall index score showed a temporary decline between 2017 and 2018, followed by a gradual recovery. This pattern implies that recent improvements in import oversight and policy refinement among major trading partners may have contributed to the recovery. The short-term dip may also reflect an increased share of imports from lower-ranked countries such as Brazil and Ukraine. This fluctuation highlights the index’s utility in monitoring changes in Korea’s aggregate exposure to food-related hazards based on shifting trade portfolios.

This study is significant in that it introduces a data-driven, systematic evaluation mechanism to complement conventional post-import inspection systems. By assessing safety levels by country and year, the APSI helps identify priority areas and provides a solid foundation for forward-looking, evidence-based policymaking. It also offers a range of practical applications: it can aid in identifying and managing high-risk countries, strengthening international collaboration in agri-food regulation, and informing communication strategies aimed at enhancing transparency and restoring consumer confidence.

Nevertheless, several methodological limitations must be acknowledged. First, aggregating diverse sub-indicators into a composite score may obscure nuanced dimensions of risk. Although equal weighting ensures transparency and replicability, it does not account for the differential impact certain variables may exert. Second, the index excludes biological hazards such as zoonoses or pathogen-related events due to a lack of consistent, internationally comparable data. Third, the model uses annual data, limiting its ability to capture short-term or seasonal fluctuations. Finally, neither the APSI nor the IFSI has yet been statistically validated using methods such as sensitivity analysis, multivariate correlation, or principal component analysis (PCA), nor have they been empirically linked to real-world food incidents or border rejection records. These limitations reduce the index’s predictive capability in complex, product-specific regulatory contexts.

Accordingly, the APSI and IFSI should be interpreted as exploratory and diagnostic tools that offer structural insight into relative import risk patterns. While the current model prioritizes transparency and policy relevance, future studies should expand its scientific rigor by incorporating empirical validation techniques, diversifying risk variables, and connecting the indices to measurable food safety outcomes. These refinements will enhance reproducibility, strengthen cross-country comparability, and enable adaptation to dynamic and commodity-specific risk profiles.

Through such improvements, the index has the potential to evolve into a comprehensive, empirically grounded policy-support tool that aligns with international standards for food safety assessment and contributes to more effective global food system governance.

## 5. Conclusions

This study developed the Agricultural Product Safety Index (APSI), a quantitative framework for evaluating the safety of imported agricultural products by country and year. The index integrates production- and distribution-related risk indicators to provide a holistic understanding of food safety within international trade systems.

Application of the index to Korea’s major agricultural trading partners from 2014 to 2021 revealed distinct cross-country differences in food safety levels. Countries with well-established agricultural infrastructure and political stability—such as Australia and the United States—demonstrated higher safety scores, while others like Brazil and Ukraine ranked lower. Korea’s overall import food safety level showed improvement after a temporary decline in 2018, reflecting the potential impact of enhanced safety management efforts.

Based on the IFSI results, the index also offers actionable insights for Korea’s import strategy. For instance, to strengthen import safety, it may be advisable to increase import volumes from high-APSI countries such as Australia. Conversely, imports from countries with lower APSI scores, such as Brazil, may require enhanced pre-import risk management and stricter inspection protocols. These findings underscore the index’s practical relevance for identifying high-risk sources and guiding regulatory attention.

While the APSI and IFSI provide structured and replicable assessments, several limitations must be acknowledged. The current version remains exploratory and has not yet undergone empirical validation using statistical techniques such as sensitivity analysis, correlation testing, or principal component analysis (PCA). In addition, the index relies on aggregated country-level data and does not reflect product-specific safety risks or short-term fluctuations. It also lacks direct empirical linkage to real-world foodborne incidents or border rejection records.

Nonetheless, the index establishes a robust foundation for future enhancements. With improved data availability and methodological refinement—including the integration of biological risk variables and time-sensitive indicators—the APSI and IFSI can evolve into more comprehensive tools. Such developments would strengthen their scientific credibility and broaden their practical utility, especially within complex and dynamic regulatory contexts.

By overcoming these limitations, the APSI has the potential to evolve into a globally adaptable instrument aligned with sustainable agriculture, resilient food systems, and long-term food security objectives.

## Figures and Tables

**Table 1:** The example of major origin selection (the case of potato).

Classification	Principle	Description
Principle 1	Applicability	Determined based on data quantitatively comparable between countries or between periods
Principle 2	Reliability	Same results produced using publicly reliable data open to anyone
Principle 3	Boundedness	Indicator determined between 0 and 1
Principle 4	Independence among indicators	No duplication between indicators
Principle 5	Representativeness	Representative countries selected based on the CR3 (Concentration Ratio)

**Table 2:** Detailed indicators of the Agricultural Product Safety Index.

Classification ^1^		Sub-Detailed Indicator	
Production indicator (PI)	Chemical factor	Pesticide use per hectare of agricultural land Quality of agricultural water	Agricultural fertilizer use per hectare of agricultural land
	Environmental factor	Abnormal temperature	Acid rain
	Other factors	Stock	Governmental investment into the agricultural sector
Distribution indicator (DI)	Other factors	Trade distance Political stability	Import non-compliance rate

^1^ Source: Indicator classification and selection based on research findings of [6] and [12].

**Table 3:** Top 3 agricultural product categories by production volume.

Classification	Sub-Detailed Indicator	Source	Description	Equation
Production index (P I)	Pesticide use per hectare of agricultural land (PU)	FAO	Reference to the data of pesticide use per hectare of agricultural land	${PUI}_{k,t}=1-\frac{{\text{PU}}_{k,t}-{\text{PU}}_{min}}{{\text{PU}}_{max}-{\text{PU}}_{min}}$
	Pesticide use per hectare of agricultural land (agricultural fertilizer use per hectare of agricultural land) (FU)	FAO	Direct calculation in reference to the data of agricultural land and use of agricultural fertilizers	${\text{FUI}}_{k,t}=1-\frac{{\text{FU}}_{k,t}-{\text{FU}}_{\text{min}}}{{\text{FU}}_{\text{max}}-{\text{FU}}_{\text{min}}}$
	Agricultural water quality (WQ)	GFSI	Reference to data of agricultural water quality released by the GFSI	${WQI}_{k,t}=\frac{{WQ}_{k,t}}{100}$
	Abnormal temperature (TA)	NOAA	Direct calculation of the total sum of the absolute values of monthly observations based on the latitude and longitude inputs of each nation’s capital city	${TAI}_{k,t}=1-\frac{{TA}_{k,t}-{TA}_{min}}{{TA}_{max}-{TA}_{min}}\;Then,\;{TA}_{k,t}=\sum |abnomal\;value$
	Stock (ST)	USDA	Reference to data of each nation’s agricultural ending stocks	${STI}_{k,t}=1-\frac{{ST}_{k,t}-{ST}_{min}}{{ST}_{max}-{ST}_{min}}$
	Governmental investment (GI)	FAO	Direct calculation of the proportion of investment into the area of ‘Water Use in Agriculture, Forestry, and Fisheries’ among the total expenditure of the central government	${GII}_{k,t}=\frac{{GI}_{k,t}-{GI}_{min}}{{GI}_{max}-{GI}_{min}}$
	Acid rain (AR)	Yale EPI	Sulfur dioxide and nitrogen oxide increase rates reflected, weighted equally at 50%	${ARI}_{k,t}=\frac{{AR}_{k,t}}{100}$
Distribution index (DI)	Trade distance (DT)	CEPII	Trade distance explicitly calculated for the destination country (South Korea)	${DTI}_{k,t}=1-\frac{{DT}_{k,t}-{DT}_{min}}{{DT}_{max}-{DT}_{min}}$
	Import non-compliance rate (UI)	Imported food Information Maru	Data of non-compliance rates for each origin of agricultural products	${UII}_{k,t}=1-\frac{{UI}_{k,t}-{UI}_{min}}{{UI}_{max}-{UI}_{min}}$
	Political stability (PS)	World Bank	Dividing the full range into 100 intervals based on calculated class width and assigning evaluation scores accordingly	${PSI}_{k,t}=\;\frac{{PS}_{max}-{PS}_{min}}{100}$

**Table 4:** Analytical data and basic statistics.

Classification	Index (Unit)	No. of Countries (Period)	No. of Observations	Average	Standard Deviation
Production index (P I)	Pesticide use per hectare of agricultural land (kg/ha)	199 countries (1990–2021)	5768	3.37	5.23
	Agricultural fertilizer use per hectare of agricultural land (kg/ha)	171 countries (2002–2021)	1904	239.97	1295.95
	Quality of agricultural water (point)	118 countries (2012–2022)	1243	41.79	39.13
	Abnormal temperature	238 countries (1990–2022)	7689	7.24	5.23
	Stock volume (1000 ton)	214 countries (1960–2023)	13,376	13,380.00	74,762.00
	Proportion of governmental investment (%)	190 countries (2001–2021)	3096	2.55	2.74
	Acid rain (point)	225 countries (1995–2022)	5404	61.83	26.91
Distribution index (DI)	Trade distance (km)	224 countries	224	9626.44	3863.08
	Import non-compliance rate (%)	132 countries (2014–2023)	837	0.41	5.20
	Political stability	198 countries (2002–2021)	3846	−0.06	0.99

**Table 5:** Selection of representative countries (^1^ based on the import share: 2014–2021).

Classification	Country	Import Share ^1^	Accumulative Share	Country	Import Share ^1^	Accumulative Share
Rank	2014			2015		
1	U.S.	0.4200	0.4200	U.S.	0.3215	0.3215
2	Brazil	0.1031	0.5231	Brazil	0.1942	0.5157
3	Australia	0.0906	0.6137	Ukraine	0.1187	0.6344
4	Ukraine	0.0873	0.7010	Australia	0.0812	0.7156
5	China	0.0525	0.7535	China	0.0572	0.7728
Rank	2016			2017		
1	U.S.	0.3594	0.3594	U.S.	0.3992	0.3992
2	Brazil	0.1533	0.5127	Brazil	0.1112	0.5104
3	Argentina	0.1022	0.6149	Australia	0.0886	0.5990
4	Australia	0.0771	0.6920	Ukraine	0.0763	0.6753
5	Ukraine	0.0689	0.7609	Argentina	0.0636	0.7389
Rank	2018			2019		
1	U.S.	0.5041	0.5041	U.S.	0.3088	0.3088
2	Australia	0.0857	0.5898	Brazil	0.1863	0.4950
3	Russia	0.0686	0.6584	Argentina	0.1412	0.6362
4	Brazil	0.0627	0.7211	Australia	0.0763	0.7125
5	China	0.0606	0.7816	China	0.0572	0.7696
Rank	2020			2021		
1	U.S.	0.3042	0.3042	U.S.	0.3122	0.3122
2	Argentina	0.1298	0.4340	Argentina	0.2010	0.5132
3	Brazil	0.1179	0.5519	Brazil	0.1111	0.6242
4	Ukraine	0.1119	0.6637	Australia	0.0839	0.7081
5	Australia	0.0710	0.7347	China	0.0526	0.7607

**Table 6:** Agricultural Product Safety Index and detailed indicators (Australia, 2014–2021).

Classification		2014	2015	2016	2017	2018	2019	2020	2021
Imported food safety index		0.8096	0.8081	0.8136	0.8124	0.8123	0.8101	0.8091	0.8087
Production indicator		0.7995	0.8066	0.8074	0.8151	0.8083	0.8072	0.8120	0.8123
	Pesticide use	0.9593	0.9572	0.9447	0.9487	0.9463	0.9457	0.9457	0.9471
	Use of agricultural fertilizers	0.9995	0.9995	0.9995	0.9996	(0.9996)	(0.9996)	(0.9996)	(0.9996)
	Quality of agricultural water	1.0000	1.0000	1.0000	1.0000	1.0000	1.0000	1.0000	1.0000
	Temperature anomaly	0.8242	0.8300	0.7883	0.8432	0.8206	0.8387	0.8868	0.9051
	Stock	0.9587	0.9575	0.9581	0.9591	0.9594	0.9554	0.9494	0.8309
	Governmental investment	0.0240	0.0229	0.0223	0.0233	0.0218	0.0247	0.0162	0.0176
	Acid rain	0.8309	0.8790	0.9388	0.9315	0.9101	0.8860	0.8860	0.8860
Distribution indicator		0.8197	0.8097	0.8197	0.8097	0.8164	0.8130	0.8062	0.8051
	Trade distance	0.5792	0.5792	0.5792	0.5792	0.5792	0.5792	0.5792	0.5792
	Import non-compliance rate	1.0000	1.0000	1.0000	1.0000	1.0000	0.9999	0.9993	0.9962
	Political stability	0.8800	0.8500	0.8800	0.8500	0.8700	0.8600	0.8400	0.8400

**Table 7:** Agricultural Product Safety Index and detailed indicators (U.S., 2014–2021).

Classification		2014	2015	2016	2017	2018	2019	2020	2021
Imported food safety index		0.7189	0.7161	0.7142	0.7150	0.7046	0.7059	0.6919	0.6916
Production indicator		0.6949	0.6826	0.6989	0.7070	0.6764	0.6988	0.6775	0.6803
	Pesticide use	0.9311	0.9295	0.9253	0.9258	0.9245	0.9245	0.9245	0.9245
	Use of agricultural fertilizers	0.9958	0.9960	0.9963	0.9963	0.9963	0.9963	0.9963	0.9963
	Quality of agricultural water	1.0000	1.0000	1.0000	1.0000	1.0000	1.0000	1.0000	1.0000
	Abnormal temperature	0.7525	0.5979	0.7580	0.7385	0.6429	0.7527	0.6675	0.7390
	Stock	0.1515	0.2187	0.1755	0.2541	0.1346	0.1702	0.0970	0.0669
	Governmental investment	0.0333	0.0363	0.0369	0.0346	0.0366	0.0477	0.0576	0.0356
	Acid rain	1.0000	1.000	1.0000	1.0000	1.0000	1.0000	1.0000	1.0000
Distribution indicator		0.7430	0.7496	0.7296	0.7229	0.7329	0.7130	0.7062	0.7030
	Trade distance	0.4389	0.4389	0.4389	0.4389	0.4389	0.4389	0.4389	0.4389
	Import non-compliance rate	1.0000	1.0000	1.0000	0.9999	0.9997	1.0000	0.9997	1.0000
	Political stability	0.7900	0.8100	0.7500	0.7300	0.7600	0.7000	0.6800	0.6700

**Table 8:** Agricultural Product Safety Index and detailed indicators (Argentina, 2016–2021).

Classification		2016	2017	2019	2020	2021
Imported food safety index		0.5894	0.6482	0.6477	0.6492	0.6451
Production indicator		0.6057	0.7233	0.7423	0.7454	0.7404
	Pesticide use	0.8710	0.8742	0.8705	0.8513	0.8519
	Use of agricultural fertilizers	0.9990	0.9989	0.9986	0.9986	(0.9986)
	Quality of agricultural water	0.0000	0.7500	0.7500	0.7500	0.7500
	Abnormal temperature	0.7493	0.6877	0.8091	0.8377	0.8062
	Stock	0.8984	0.9061	0.8995	0.9073	0.9082
	Governmental investment	0.0274	0.0303	0.0147	0.0190	0.0140
	Acid rain	0.6948	0.8156	0.8537	0.8537	0.8537
Distribution indicator		0.5731	0.5731	0.5531	0.5531	0.5498
	Trade distance	0.0093	0.0093	0.0093	0.0093	0.0093
	Import non-compliance rate	1.0000	1.0000	1.0000	1.0000	1.0000
	Political stability	0.7100	0.7100	0.6500	0.6500	0.6400

**Table 9:** Agricultural Product Safety Index and detailed indicators (Ukraine, 2014–2020).

Classification		2014	2015	2017	2020
Imported food safety index		0.6147	0.6171	0.6319	0.6454
Production indicator		0.5989	0.6003	0.6232	0.6002
	Pesticide use	0.9386	0.9489	0.9697	0.9809
	Use of agricultural fertilizers	(0.9969)	(0.9969)	(0.9969)	0.9952
	Quality of agricultural water	0.0000	0.0000	0.0000	0.0000
	Abnormal temperature	0.5796	0.5179	0.5820	0.2564
	Stock	0.9786	0.9815	0.9797	0.9771
	Governmental investment	0.0543	0.0346	0.0536	0.0456
	Acid rain	0.6441	0.7224	0.7808	0.9464
Distribution indicator		0.6305	0.6338	0.6405	0.6905
	Trade distance	0.6315	0.6315	0.6315	0.6315
	Import non-compliance rate	(1.0000)	1.0000	1.0000	1.0000
	Political stability	0.2600	0.2700	0.2900	0.4400

**Table 10:** Agricultural Product Safety Index and detailed indicators (Brazil, 2014–2021).

Classification		2014	2015	2016	2017	2018	2019	2020	2021
Imported food safety index		0.5923	0.5808	0.5707	0.578217	0.5792	0.5733	0.5963	0.5959
Production indicator		0.6130	0.6067	0.5897	0.6115	0.6100	0.6184	0.6444	0.6468
	Pesticide use	0.7949	0.7902	0.7838	0.7846	0.7726	0.7455	0.7213	0.7104
	Use of agricultural fertilizers	(0.9955)	(0.9955)	(0.9955)	(0.9955)	(0.9955)	(0.9955)	(0.9955)	(0.9955)
	Quality of agricultural water	0.5000	0.5000	0.5000	0.5000	0.5000	0.5000	0.5000	0.5000
	Abnormal temperature	0.8981	0.7491	0.7592	0.8278	0.9037	0.7532	0.8495	0.8601
	Stock	0.5952	0.7322	0.5480	0.5814	0.4413	0.5997	0.7152	0.7289
	Governmental investment	0.0446	0.0382	0.0410	0.0346	0.0318	0.0311	0.0251	0.0289
	Acid rain	0.4627	0.4421	0.5006	0.5570	0.6253	0.7039	0.7039	0.7039
Distribution indicator		0.5716	0.5549	0.5516	0.5449	0.5483	0.5283	0.5483	0.5449
	Trade distance	0.0648	0.0648	0.0648	0.0648	0.0648	0.0648	0.0648	0.0648
	Import non-compliance rate	1.0000	1.0000	1.0000	1.0000	1.0000	1.0000	1.0000	1.0000
	Political stability	0.6500	0.6000	0.5900	0.5700	0.5800	0.5200	0.5800	0.5700

**Table 11:** Process and results of the Agricultural Product Safety Index calculation.

Classification	Agricultural Product Safety Index	Import Share	Imported Food Safety Index
Year	2014		
Australia	0.8096	12.9%	0.699
U.S.	0.7189	59.9%	
Ukraine	0.6147	12.4%	
Brazil	0.5923	14.7%	
Year	2015		
Australia	0.8081	11.3%	0.673
U.S.	0.7161	44.9%	
Ukraine	0.6171	16.6%	
Brazil	0.5808	27.1%	
Year	2016		
Australia	0.8136	11.1%	0.675
U.S.	0.7142	51.9%	
Argentina	0.5894	14.8%	
Brazil	0.5707	22.2%	
Year	2017		
Australia	0.8124	12.0%	0.692
U.S.	0.7150	54.0%	
Argentina	0.6482	8.6%	
Ukraine	0.6319	10.3%	
Brazil	0.5782	15.0%	
Year	2018		
Australia	0.8123	11.9%	0.640
U.S.	0.7046	69.9%	
Brazil	0.5792	8.7%	
Year	2019		
Australia	0.8101	10.7%	0.671
U.S.	0.7059	43.3%	
Argentina	0.6477	19.8%	
Brazil	0.5733	26.1%	
Year	2020		
Australia	0.8091	9.7%	0.673
U.S.	0.6919	41.4%	
Argentina	0.6492	17.7%	
Ukraine	0.6454	15.2%	
Brazil	0.5963	16.0%	
Year	2021		
Australia	0.8087	11.8%	0.677
U.S.	0.6916	44.1%	
Argentina	0.6451	28.4%	
Brazil	0.5959	15.7%	

## Data Availability

The original contributions presented in the study are included in the article. Further inquiries can be directed to the corresponding authors.
